# Germacrone alleviates neurological deficits following traumatic brain injury by modulating neuroinflammation and oxidative stress

**DOI:** 10.1186/s12906-020-03175-0

**Published:** 2021-01-05

**Authors:** Sujing Zhuang, Baogui Liu, Shifeng Guo, Yanzhong Xue, Lin Wu, Shiqi Liu, Chunling Zhang, Xiuyan Ni

**Affiliations:** 1 Department of Neurology, Linyi Central Hospital, Linyi, 276400 Shandong China; 2 Department of Anesthesiology, Linyi Central Hospital, Linyi, 276400 Shandong China; 3 Department of Intensive Care Unit 2, Affiliated Hospital of Jining Medical University, Jining, 272000 Shandong China; 4 Department of Gastrointestinal Surgery, Affiliated Hospital of Jining Medical University, Jining, 272000 Shandong China; 5 Department of Radiotherapy, Linyi Central Hospital, Linyi, 276400 Shandong China

**Keywords:** Germacrone, Traumatic brain injury, NF-κB, Inflammation

## Abstract

**Background:**

Germacrone (GM) is a terpenoid compound which is reported to have anti-inflammatory and anti-oxidative effects. However, its role in treating traumatic brain injury (TBI) remains largely unknown.

**Methods:**

Male C57BL/6 mice were divided into the following groups: control group, TBI group [controlled cortical impact (CCI) model], CCI + 5 mg/kg GM group, CCI + 10 mg/kg GM group and CCI + 20 mg/kg GM group. GM was administered via intraperitoneal injection. The neurological functions (including motor coordination, spatial learning and memory abilities) and brain edema were measured. Nissl staining was used to detect the neuronal apoptosis. Colorimetric assays and enzyme linked immunosorbent assay (ELISA) kits were used to determine the expression levels of oxidative stress markers including myeloperoxidase (MPO), malondialdehyde (MDA) and superoxide dismutase (SOD), as well as the expressions of inflammatory markers, including tumor necrosis factor α (TNF-α), interleukin-1β (IL-1β) and interleukin-6 (IL-6). Additionally, protein levels of Nrf2 and p-p65 were detected by Western blot assay.

**Results:**

GM significantly ameliorated motor dysfunction, spatial learning and memory deficits of the mice induced by TBI and it also reduced neuronal apoptosis and microglial activation in a dose-dependent manner. Besides, GM treatment reduced neuroinflammation and oxidative stress compared to those in the CCI group in a dose-dependent manner. Furthermore, GM up-regulated the expression of antioxidant protein Nrf2 and inhibited the expression of inflammatory response protein p-p65.

**Conclusions:**

GM is a promising drug to improve the functional recovery after TBI via repressing neuroinflammation and oxidative stress.

## Background

Traumatic brain injury (TBI) is one of the common causes of disability and death. Patients with TBI often have cognitive dysregulation, dyskinesia, memory impairment and other neurological problems [1]. TBI has both a direct mechanical effect (primary injury) and an indirect effect caused by a complex pathological cascade (secondary injury). Subsequent biological processes trigger the secondary damage, such as excitotoxicity, which gives rise to many biological events, including the delayed afterdepolarization, inflammation and neuronal apoptosis around the lesion [2]. Preventing or ameliorating secondary brain injury has great significance to improve the prognosis of the patients with TBI.

Neuronal inflammation caused by activation of microglia figures prominently in secondary injury in TBI [3, 4], which can result in post-traumatic epilepsy and increase the risk of cognitive impairment and other sequelae [5]. After TBI, microglia are activated, and peripheral macrophages migrate to the injured site and secrete a large number of inflammatory cytokines, such as tumor necrosis factors, interleukins and interferons, which contribute to inflammatory response and neuronal apoptosis [6]. NF-κB signaling plays a key role in regulating the expression and activation of NLRP1 and NLRP3 inflammasomes in neurons and brain tissue [7]. Besides, inhibition of NF-κB signaling pathway can prevent acute brain injury by inhibiting excessive microglial activation and promoting neuronal survival [8]. In addition, excessive activation of microglia produces reactive oxygen species (ROS). Excessive ROS interferes with the normal structure and the function of proteins and lipids, inducing DNA damage and cellular apoptosis [9]. Nuclear factor, erythroid 2 like 2 (Nrf2) is a basic leucine zipper (bZIP) transcription factor and a crucial regulator for inducing the expressions of antioxidant proteins and preventing oxidative damage [10–12]. For example, Nrf2 participates in the brain’s defense mechanism to protect itself from ischemia-reperfusion injury [11].

Germacrone (GM) is one of the main bioactive components extracted from *Curcuma zedoaria Roscoe*, which has a wide range of biological effects such as anti-inflammation, anti-oxidation and anti-tumor functions [13–15]. It is reported that GM blocks the progression of arthritis by regulating Th1/Th2 balance and inhibiting NF-κB signaling [16]. What’s more, GM can markedly reduce the expressions of the pro-inflammatory cytokines IL-6 and TNF-α, while promoting the expressions of the anti-inflammatory mediators TGF-β and IL-10, and has a protective effect on the acute lung injury caused by lipopolysaccharide in newborn rats [17]. Additionally, GM reduces neurological injury caused by cerebral ischemia-reperfusion in rats through antioxidant and anti-apoptotic mechanisms [15]. However, its neuroprotective functions in TBI have not been explored.

The purpose of this study was to study the neuroprotective effects of GM on a mice model with TBI, and to investigate the underlying molecular mechanisms.

## Methods

### Animal model

Male C57BL/6 mice aged from 7 to 8 weeks (weight about 20–23 g) were purchased from the Model Animal Research Center of Nanjing University (Nanjing, China). The mice were raised in a controlled environment (12-h light / 12-h dark cycle, 22 ± 2 °C, 40–60% humidity) and supplied with enough food and water. All of the procedures in animal experiments in this work were approved by the Institutional Animal Care and Use Committee of Affiliated Hospital of Jining Medical University.

**Fig. 1:**
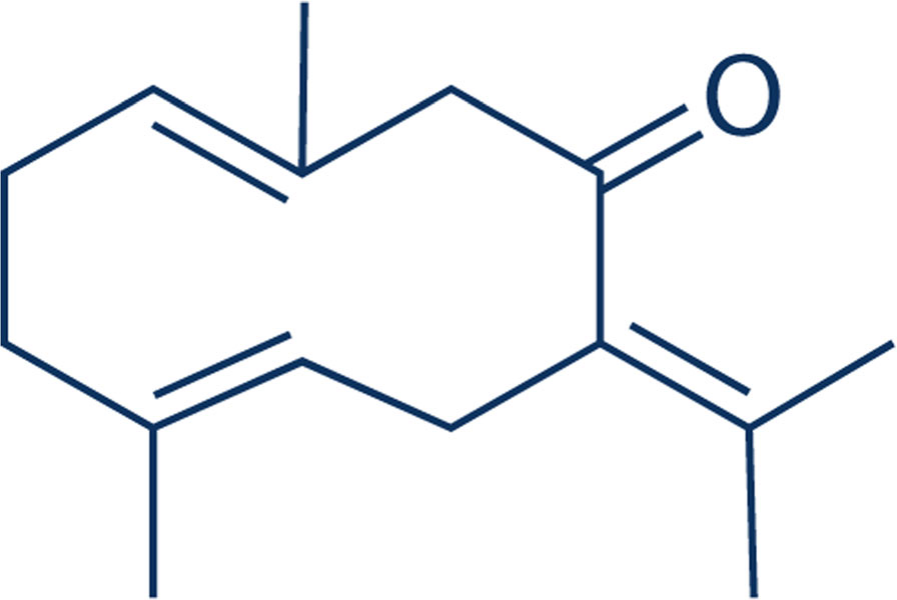
The molecular structure of GM

Fifty mice were randomly divided into 5 groups (10 mice per group): 1. Control (sham) group; 2. TBI (controlled cortical impact [CCI]) group; 3. CCI + 5 mg/kg GM group (CCI mice treated with 5 mg/kg GM), 4. CCI + 10 mg/kg GM group (CCI mice treated with 10 mg/kg GM) and 5. CCI + 20 mg/kg GM group (CCI mice treated with 20 mg/kg GM). 2 h after CCI, in GM treatment groups, the mice were intraperitoneally injected with 5, 10 and 20 mg/kg GM (Fig.1, Sigma-Aldrich, St. Louis., MO, USA). Mice in the CCI and control groups were administrated with normal saline. The dosing, delivery route and treatment regimens were based on previous studies [16, 18]. There was no significant difference in body weight and blood glucose in GM-treated mice compared with those in the mice in CCI group and control group (data not shown). After GM treatment, some of the mice (4 mice per group) were sacrificed with asphyxia (20% CO_2_ for 10 min), and the brain tissues were collected for subsequent examinations, and the neurological functions were evaluated with the other mice (6 mice per group).

TBI animal model was established by CCI. In brief, the surgery was performed after the mice were anaesthetized with isoflurane (2%). After craniotomy, CCI was conducted with a stainless-steel pointed piston with a diameter of 3 mm in a stereotactic impactor (Leica, Wetzlar, Germany), centered at 2 mm posterior to bregma and 2.5 mm left of midline [19]. The piston speed was set at 5 m/s and the depth of 1.5 mm, and a moderate cortical contusion was produced in the left lobe of the brain [20]. In the sham group, the mice were anesthetized with isoflurane and then craniotomy was performed, without traumatic induction.

### Rota-rod test and Morris water maze assay

The rota-rod assay was used to assess motor function and the balance of the mice. The day before the experiment began, the mice were placed on a rotating rod at a constant speed of 4 rpm in order to get accustomed to the LE8200 rota-rod device (Letica Scientific Instruments, Spain). After the experiment began, each mouse was placed on the accelerating rotating rod (at a speed of 4 to 40 rpm in 5 min). The experiment was repeated for three times for each animal, and the average time during which an animal could maintain itself on the device was recorded.

Morris water maze assay was used to evaluate the spatial learning and memory of the mice as previously described [21]. Briefly, the mice were placed in a black round tank (with a diameter of 150 cm) filled with water. The mice were trained to swim to a hidden platform four times a day for seven consecutive days before the experiment. If the mice found the platform in 60 s and stayed on it for 10 s, they would be taken out of the swimming pool. If the mice could not find the platform within 60 s, they were gently guided to the platform and allowed to stay on the platform for another 20 s. After the experiment began, the platform was removed. The mice were allowed to swim freely for 60 s. The number of times that the mice passed through the platform area and the time spent in the quadrant with the platform area were recorded as the scores.

### Hematoxylin and eosin (HE) staining and Nissl’s staining

After the mice were deeply anesthetized, the chest was opened and the hearts were perfused with normal saline until colorless liquid flowed from right auricle. Then the mice were perfused with 4% paraformaldehyde, and the brains of the mice were obtained and fixed in 10% formaldehyde for 24 h. After the specimens were embedded in paraffin and sliced (into 4 mm thick sections), the sections containing the hippocampal tissues were used for HE staining, and the sections were observed under a light microscope (Nikon, Tokyo, Japan).

After dewaxing and rehydration, the sections were stained with Nissl staining solution (Beyotime, Shanghai, China) for 30 min at 60 °C, dehydrated with ethanol, made transparent with xylene and sealed with neutral gum, and the sections were observed and photographed under a microscope (Nikon, Tokyo, Japan).

### Evaluation of brain edema

After TBI, the hemisphere with the lesion of the mice was removed and weighed to obtain a wet weight. Then the brain tissues were placed in an oven and dried for 24 h, and then the dried brain tissues were weighed to obtain a dry weight. Brain water content (%) was calculated according to the formula: (wet weight-dry weight)/wet weight × 100%.

### Evaluation of inflammatory responses and oxidative stress

The brain tissue of the mice was homogenized on ice and the supernatant was collected by centrifugation at 2500×g for 20 min. Then the concentrations of inflammatory cytokines TNF-α, IL-1β and IL-6 in brain tissues were measured with enzyme-linked immunosorbent assay (ELISA) method according to the manufacturer’s instructions using corresponding ELISA kit (Solarbio, Beijing, China). Colorimetric methods were used to determine the activities of myeloperoxidase (MPO), malondialdehyde (MDA) and superoxide dismutase (SOD). The kits for detecting the above oxidative stress indicators were purchased from Solarbio (Beijing, China).

### Quantitative real-time polymerase chain reaction (qRT-PCR)

Total RNA was isolated using TRIzol reagent (Invitrogen, Carlsbad, CA) in line with the manufacturer’s instructions. The RNA was reversely transcribed into cDNA using PrimeScript™ RT reagent Kit with gDNA Eraser (Takara, Dalian, China). The SYBR® Premix Ex Taq™ II (Takara, Dalian, China) was used to perform qRT-PCR assay. Relative quantification of the genes was performed using 2^-ΔΔCt^ method and glyceraldehyde 3-phosphate dehydrogenase (GAPDH) was used as the reference gene. Specific primers were as follows: CD11b primers: forward: 5′-ATGGACGCTGATGGCAATACC-3 ‘and reverse: 5’-TCCCCATTCACGTCTCCCA-3′; CD16 primers: forward: 5′-CAGAATGCACACTCTGGAAGC-3 ‘and reverse: 5’-GGGTCCCTTCGCACATAG-3 ‘; TNF-α primers: forward: 5’-CCCTCACACTCAGATCATCTTCT-3′ and reverse: 5′-GCTACGACGTGGGCTACAG-3 ‘; IL-6 primers: forward: 5’-TAGTCCTTCCTCTACCCCAATTTCC-3′ and reverse: 5′-TGGTCCTTAGCCACTCCTTCTC -3 ‘; IL-1β primers: forward: 5’-GCAACTGTTCCTGAACTCAACT-3′ and reverse: 5′-ATCTTTTGGGGTCCGTCAACT-3 ‘; GAPDH primers: forward: 5’-TCATCCCAGAGCTGAACG-3′ and reverse: 5′-TCATACTTGGCAGGTTTCTCC − 3 ‘.

### Western blot assay

Proteins from brain tissues (homogenized) were extracted using RIPA buffer (Biosharp, Hefei, China) and quantified using a bicinchoninic acid kit (Biosharp, Hefei, China). The extractive was heated in boiling water for 10 min, then separated by 10% sodium dodecyl sulfate-polyacrylamide gel electrophoresis and transferred to a PVDF membrane (Millipore, Bedford, MA, USA). The membrane was blocked with 5% skim milk and then incubated with primary antibodies anti-Nrf2 (1: 1000; ab62352; Abcam), anti-NF-κB phosphorylated p65 (p-p65) (S536) (1: 1000; ab86299; Abcam), anti-CD16 (1: 1000; ab203883; Abcam), anti-CD11b (1: 1000; ab13357; Abcam) and anti-β-actin (1: 1000; ab8226; Abcam), respectively, followed by being gently shaken at 4 °C overnight. The membranes were then rinsed with TBST and incubated with the corresponding horseradish peroxidase (HRP)-conjugated secondary antibodies (Proteintech, Wuhan, China) for 1 h at room temperature. Finally, electrochemiluminescence kit (Biosharp, Hefei, China) was used for developing the protein bands. The gray value of each band was analyzed with the software ImageJ (NIH, Bethesda, MD, USA).

### Statistical analysis

The results in this study were expressed as mean ± standard deviation (x ± s) and SPSS statistical software (version 22.0, Chicago, IL, USA) was used to analyze the data. The data in two groups were compared using *t*-test and the data in multiple groups were compared using one-way ANOVA. GraphPad Prism 6.0 (GraphPad Software, San Diego, CA, USA) was used for graphing. *P* < 0.05 was considered statistically significant.

## Results

### GM ameliorated neurological dysfunction and neurological injury in CCI mice

**Fig. 2:**
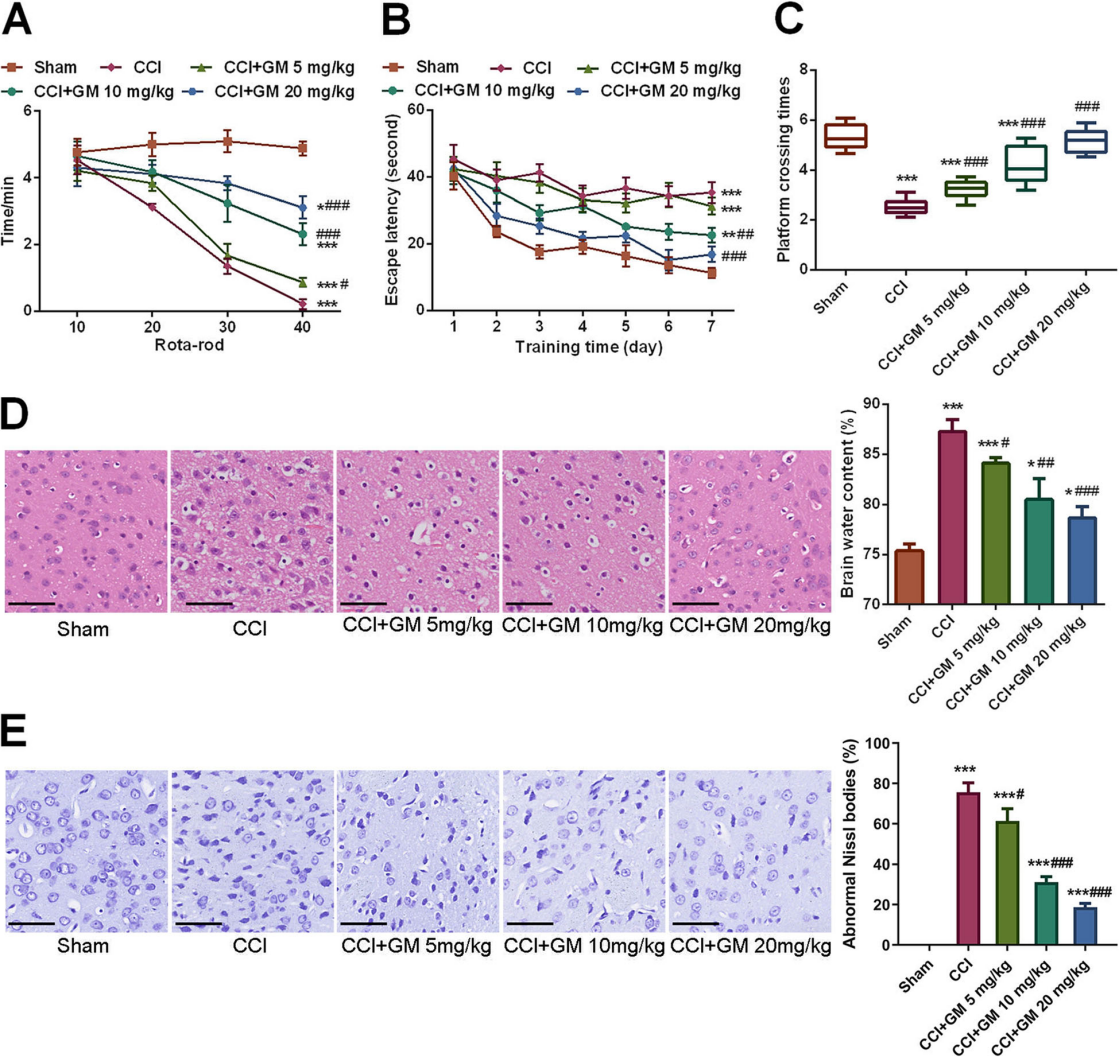
GM ameliorated the brain injury caused by CCI in mice. **a** Rota-rod test was used to test the motor function of mice in each group. **b**, **c** Morris water maze test was used to detect the spatial learning and memory functions of mice in each group. **d** The brain tissue of mice in each group was examined with HE staining (left) and brain edema was measured by dry-wet method (right). (Bar = 50 μm). **e** The brain tissue of mice was stained with Nissl staining solution and the number of abnormal Nissl body was counted. (Bar = 50 μm). Sham: Control group; CCI: TBI model group; CCI + GM 5 mg/kg: CCI mice treated with 5 mg/kg GM group; CCI + GM 10 mg/kg: CCI mice treated with 10 mg/kg GM group; CCI + GM 20 mg/kg: CCI mice treated with 20 mg/kg GM group; * *P* < 0.05, ** *P* < 0.01 and *** *P* < 0.001 versus the Sham group; # *P* < 0.05, ## *P* < 0.01 and ### *P* < 0.001 versus the CCI group

To evaluate the neuroprotective effect of GM on TBI in mice, the mice model with TBI was treated with 5, 10 or 20 mg/kg GM. The motor function, spatial learning and memory abilities of mice were detected by rota-rod test and Morris water maze assay. The results depicted that compared with in sham group, the above functions were decreased dramatically in mice with TBI while GM remarkably improved the motor function, spatial learning and memory functions in a dose-dependent manner (vs. CCI group, Fig. 2a-c). HE staining of hippocampal tissues unearthed that GM reduced the edema in the extracellular space and surrounding blood vessels, and wet-dry method also indicated that cerebral edema of the mice was also markedly ameliorated by GM treatment (vs. CCI group, Fig. 2d). Besides, the results of Nissl staining manifested that, in the control group, neurons are with clear cell outline, and the cellular structure was intact with abundant cytoplasm; in CCI group, damaged neuron was present with deformation and condensation of cytoplasm and nuclei; however, GM treatment partly reversed the pathological changes (vs. CCI group, Fig. 2e). These findings demonstrated that GM could ameliorate the neurological injury caused by CCI.

### GM reduced microglial activation in CCI mice

**Fig. 3:**
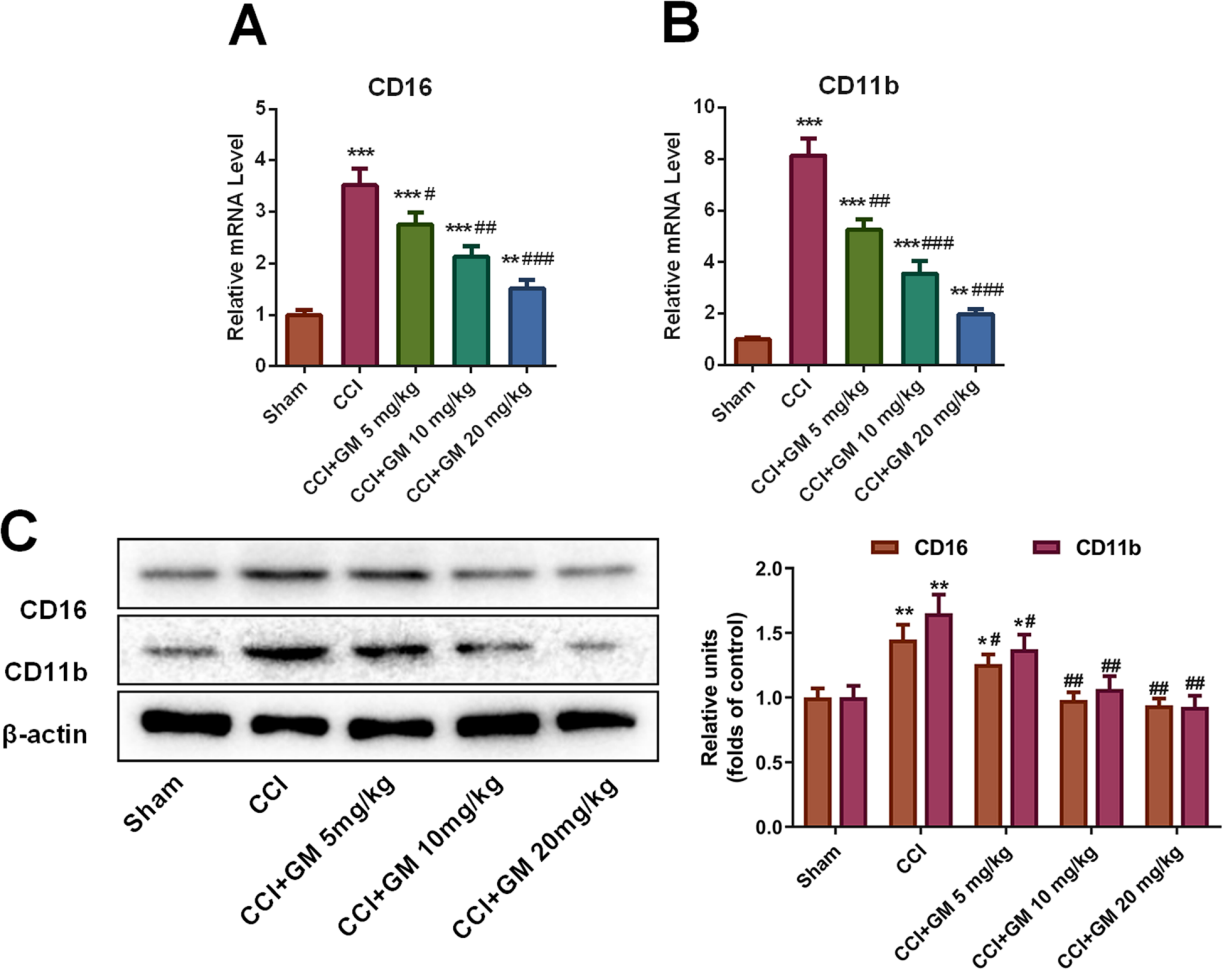
GM reduced microglial activation in brain tissue of mice with TBI. **a**, **b** The mRNA expressions of microglial activation markers CD16 and CD11b in the brain tissue of mice in each group were detected by qRT-PCR assay. **c** Western blot assay was used to detect the expressions of microglia activation markers CD16 and CD11b in the brain tissue of mice in each group. Sham: Control group; CCI: TBI model group; CCI + GM 5 mg/kg: CCI mice treated with 5 mg/kg GM group; CCI + GM 10 mg/kg: CCI mice treated with 10 mg/kg GM group; CCI + GM 20 mg/kg: CCI mice treated with 20 mg/kg GM group;* *P* < 0.05, ** *P* < 0.01 and *** *P* < 0.001 versus the sham group; # *P* < 0.05, ## *P* < 0.01 and ### *P* < 0.001 versus the CCI group

Reportedly, microglial activation is closely related to the secondary injury after TBI [3, 4]. In this study, the mRNA and protein levels of microglial activation markers CD16 and CD11b in the brain tissues of the mice in each group were detected by qRT-PCR and Western blot assays. The results showed that microglia were activated in the brain tissue of mice with TBI: the mRNA and protein expression levels of the two markers of activated microglia, CD16 and CD11b, were significantly increased in CCI group, while GM treatment observably decreased the expressions of CD16 and CD11b (Fig. 3a-c). These results suggested that GM inhibited microglial activation after TBI.

### GM reduces the levels of pro-inflammatory cytokines in the brain tissues of the mice with TBI

**Fig. 4:**
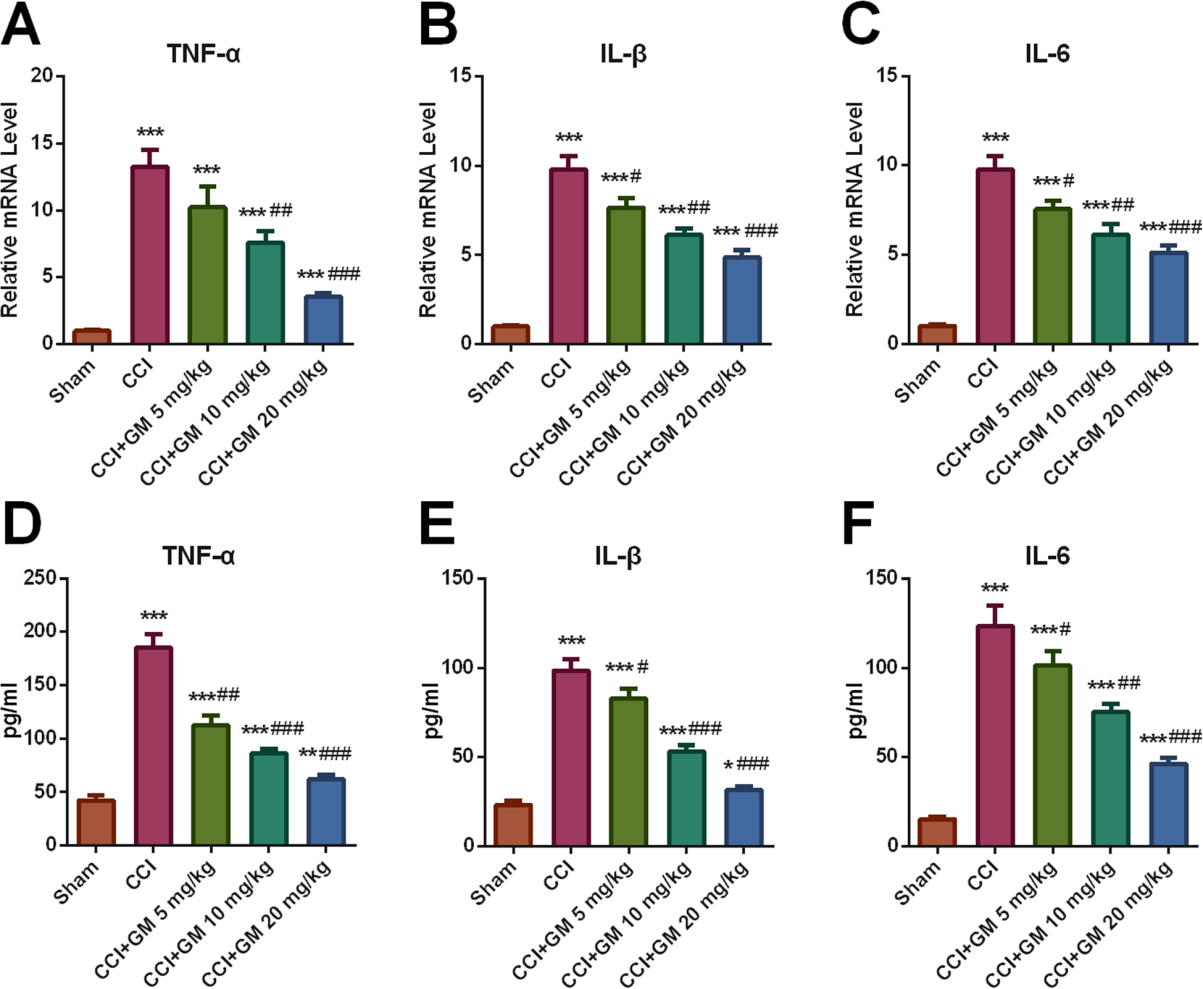
GM increased the expression level of pro-inflammatory cytokines in CCI-treated mice. **a**-**c** qRT-PCR assay was used to detect the relative expressions of inflammatory factors TNF-α, IL-1β and IL-6 mRNA in the brain tissue of mice in each group. **d**-**f** ELISA kit was used to detect the contents of inflammatory factors TNF-α, IL-1β and IL-6 in the brain tissue of mice in each group. Sham: Control group; CCI: TBI model group; CCI + GM 5 mg/kg: CCI mice treated with 5 mg/kg GM group; CCI + GM 10 mg/kg: CCI mice treated with 10 mg/kg GM group; CCI + GM 20 mg/kg: CCI mice treated with 20 mg/kg GM group; * *P* < 0.05, ** *P* < 0.01 and *** *P* < 0.001 versus the sham group. # *P* < 0.05, ## *P* < 0.01 and ### *P* < 0.001 versus the CCI group

Neuroinflammation after TBI is closely associated with microglial activation [6]. To observe the difference of pro-inflammatory cytokines’ expressions of the mice in different groups, qRT-PCR and ELISA assays were performed. qRT-PCR assay verified that after CCI, expressions of TNF-α, IL-6 and IL-1β mRNA were markedly increased (vs. sham group), and the GM treatment (5, 10 and 20 mg/kg) notably repressed the expressions of TNF-α, IL-1β and IL-6 in a dose-dependent manner (vs. CCI group, Fig. 4a-c). Consistently, ELISA unmasked that after TBI, TNF-α, IL-6 and IL-1β expressions in the brain tissues of the mice were dramatically increased (vs. sham group) and gradually decreased with the treatment of higher doses of GM (vs. CCI group, Fig. 4d-f). Therefore, it was concluded that GM could suppress the inflammatory response in brain after TBI.

### GM reduced oxidative stress in CCI mice

**Fig. 5:**
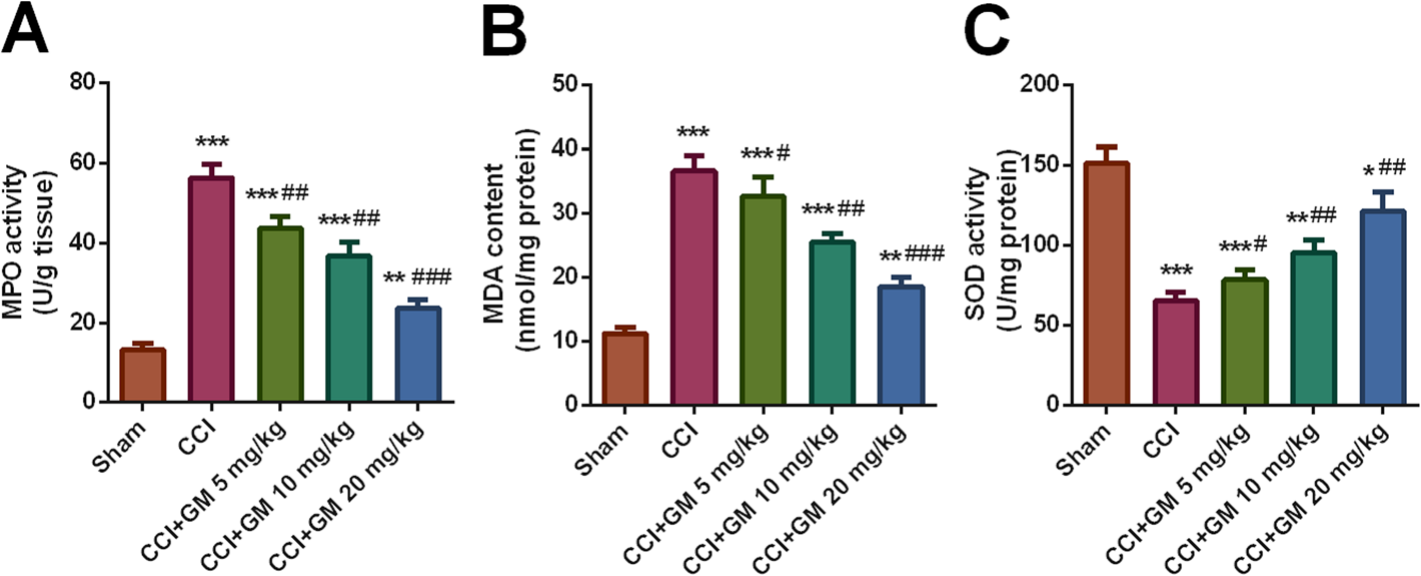
GM reduced oxidative stress response in CCI-treated mice. **a**-**c** . The activity of MPO, the content of MDA and the activity of SOD in the brain tissue of mice in each treatment group were detected with corresponding kits. Sham: Control group; CCI: TBI model group; CCI + GM 5 mg/kg: CCI mice treated with 5 mg/kg GM group; CCI + GM 10 mg/kg: CCI mice treated with 10 mg/kg GM group; CCI + GM 20 mg/kg: CCI mice treated with 20 mg/kg GM group; * *P* < 0.05, ** *P* < 0.01 and *** *P* < 0.001 versus the sham group; # *P* < 0.05, ## *P* < 0.01 and ### *P* < 0.001 versus the CCI group

To further expound the effect of GM on oxidative stress, the oxidative stress in the brain of mice in different groups was analyzed. Compared with the sham group, MPO activity and MDA level in the brain tissue of mice treated with CCI were significantly enhanced; MPO and MDA were markedly inhibited after GM treatments (vs. CCI group, Fig. 5a-b). Moreover, compared with in the sham group, SOD activity in the brain tissues of the mice in the CCI group was significantly reduced; conversely, GM could markedly increase the expression level of SOD in CCI mice (vs. CCI group, Fig. 5c).

### GM induced anti-oxidative and anti-inflammatory responses via regulating Nrf2 and NF-κB pathways

**Fig. 6:**
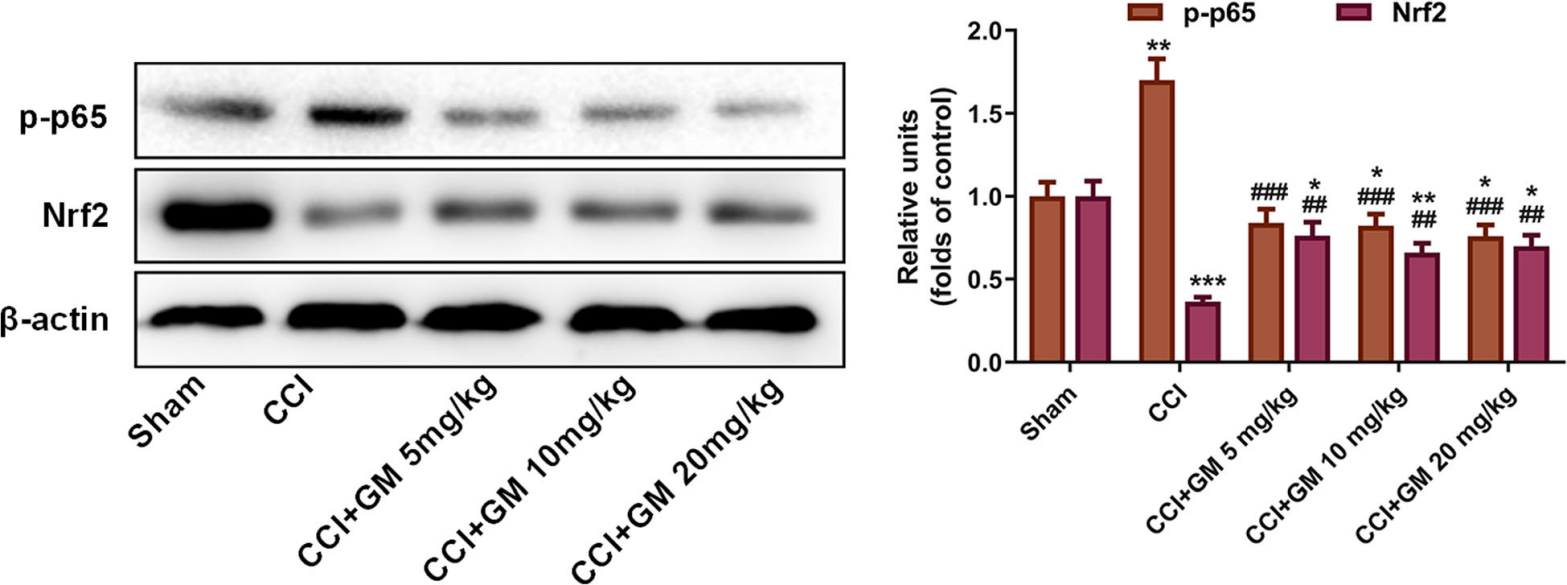
GM induced anti-oxidative and anti-inflammatory responses via regulating Nrf2 and NF-κB pathways. In each group, the protein levels of Nrf2 and NF-κB p-p65 in brain tissue of CCI mice were detected by Western blot assay. Sham: Control group; CCI: TBI model group; CCI + GM 5 mg/kg: CCI mice treated with 5 mg/kg GM group; CCI + GM 10 mg/kg: CCI mice treated with 10 mg/kg GM group; CCI + GM 20 mg/kg: CCI mice treated with 20 mg/kg GM group; * *P* < 0.05, ** *P* < 0.01 and *** *P* < 0.001 versus the sham group; ## *P* < 0.01 and ### *P* < 0.001 versus the CCI group

The above results showed that GM could remarkably inhibit the inflammation and oxidative stress induced by CCI. NF-κB exerts a crucial role in regulating downstream inflammatory cytokines and mediators; Nrf2 is an essential endogenous transcription factor in cells to defend against oxidative stress [7–12]. Western blot assay indicated that compared with in the sham group, the expression of p-p65 in mice of CCI group was increased, suggesting that NF-κB signaling was activated, while GM treatment could significantly inhibit the excessive expression of p-p65 (vs. CCI group, Fig. 6). Western blot assay also suggested that GM could induce the expression of Nrf2 to exert antioxidant effects (vs. CCI group, Fig. 6). Therefore, we supposed that GM could probably exert anti-inflammatory and anti-oxidative effects via regulating the NF-κB and Nrf2 pathways.

## Discussion

Inflammatory response and oxidative stress are considered to be the main biological events participating in the secondary brain injury caused by TBI [22]. In this study, we found that GM treatment could ameliorate the secondary brain injury with a mice model with TBI, accompanied by better motor function and spatial memory, reduced brain edema, less severe neuroinflammation and oxidative stress. Besides, it was demonstrated that GM could probably suppress NF-κB signaling and activate Nrf2 pathway. Our data suggested that GM was a promising drug to prevent or ameliorate the secondary brain injury induced by TBI.

In a rat model with cerebral ischemia, GM treatment attenuates ischemia/reperfusion-induced the brain injury of animals: GM treatment reduces MDA expression level and promotes the activities of glutathione, SOD and glutathione peroxidase; GM treatment also decreases the expressions of caspase-3 and Bax, and increases the expression of Bcl-2 [15]. Another study proves that GM treatment can ameliorate the brain injury of rats caused by middle cerebral artery occlusion/reperfusion, via regulating PI3K/Akt/mTOR signaling and repressing autophagy [23]. It is well known that hippocampal neuron death is closely linked to the dysfunction of learning and memory [24]. In the present work, we demonstrated that GM could attenuate the edema and neuron injury of hippocampal tissues induced by TBI, accompanied by improved spatial learning and memory abilities. Additionally, after the mice with TBI were treated with GM, the motor function was also improved. Our data further validated that GM had neuroprotective properties, which is consistent with the previous reports [15, 23].

Microglia are the smallest glial cells, which are distributed throughout the central nervous system, accounting for about 5–10% of total glial cells [25]. The activation of microglia and the release of inflammatory cytokines are key factors in the inflammatory response in nervous system after injury [26]. In this study, we found that GM could remarkably suppress the expressions of microglial activation markers CD16 and CD11b, suggesting GM could repress the activation of microglial cells. Our results also authenticated that GM could significantly inhibit the expressions of inflammatory cytokines, including TNF-α, IL-6 and IL-1β, thus suppressing the inflammatory response. NF-κB is one of the key regulators of inflammation after brain injury [27, 28]. Therefore, in this study, the expression of NF-κB was further analyzed by Western blot assay. The results manifested that NF-κB was over-activated in the brain tissues of the mice in CCI group, but the phosphorylation of p65 was markedly blocked by GM treatment. These results indicated that GM might have a neuroprotective effect via inhibiting the activation of NF-κB signaling.

Oxidative stress features prominently in the occurrence of secondary injuries [29]. In neurons, the increase of ROS triggers DNA damage and neuronal apoptosis. Nrf2 modulates hundreds of genes, of which many are involved in regulating ferroptosis, metabolism of glutathione, iron and lipids homeostasis and mitochondrial function, and the activation of Nrf2/ARE signaling, which up-regulates the expressions of antioxidant genes, such as HO-1 and NQO1, shows neuroprotective functions in animal models of cerebral ischemia, Parkinson’s disease, Alzheimer’s disease and amyotrophic lateral sclerosis [30, 31]. Activating Nrf2 signaling is a promising strategy to attenuate the secondary injury after TBI. For example, valproate treatment promotes the activity of Nrf2/ARE pathway and inhibits the autophagy of neurons after TBI, alleviating neurological impairment, including brain edema and neuronal apoptosis in a rat model [32]. In this study, we found that content of MDA and MPO activity in the brain tissue of GM-treated mice was dramatically reduced and the SOD activity was dramatically increased, suggesting that GM inhibited the oxidative stress response induced by TBI. Further analysis of the Nrf2 protein revealed that GM treatment remarkably facilitated the expression of Nrf2, indicating that GM-mediated anti-oxidative effects might be caused by the activation of Nrf2 pathway.

## Conclusion

In summary, current research implies that GM can ameliorate the neurological dysfunction, inflammation and oxidative stress induced by TBI, probably via inhibiting NF-κB signaling activating Nrf2 pathway. This study not only explores the new mechanism of GM improving TBI injury but also provides clues for the therapeutic strategy of TBI.

## Supplementary Information

**Additional file 1.** Supplementary materials Original image of Western blot experiment in this study.

## Data Availability

The data used to support the findings of this study are available from the corresponding author upon request.

## Abbreviations

GM: Germacrone
TBI: Traumatic brain injury
CCI: Controlled cortical impact
MPO: Myeloperoxidase
MDA: malondialdehyde
SOD: Superoxide dismutase
TNF-α: Tumor necrosis factor α
IL-1β: Interleukin-1β
IL-6: Interleukin-6
ROS: Reactive oxygen species
Nrf2: Nuclear factor carotenoid 2 related factor 2
HE: Hematoxylin and eosin
qRT-PCR: Quantitative real-time polymerase chain reaction
GAPDH: Glyceraldehyde 3-phosphate dehydrogenase
