# Supplementary material for: Germacrone alleviates neurological deficits following traumatic brain injury by modulating neuroinflammation and oxidative stress
Source: BMC Complement Med Ther. 2021 Jan 5;21:6. doi: 10.1186/s12906-020-03175-0 (PMC7786997; doi:10.1186/s12906-020-03175-0)
Supplement: Supplementary file 1 — Additional file 1. Supplementary materials Original image of Western blot experiment in this study. [file 12906_2020_3175_MOESM1_ESM.docx]

**Figure 3C**


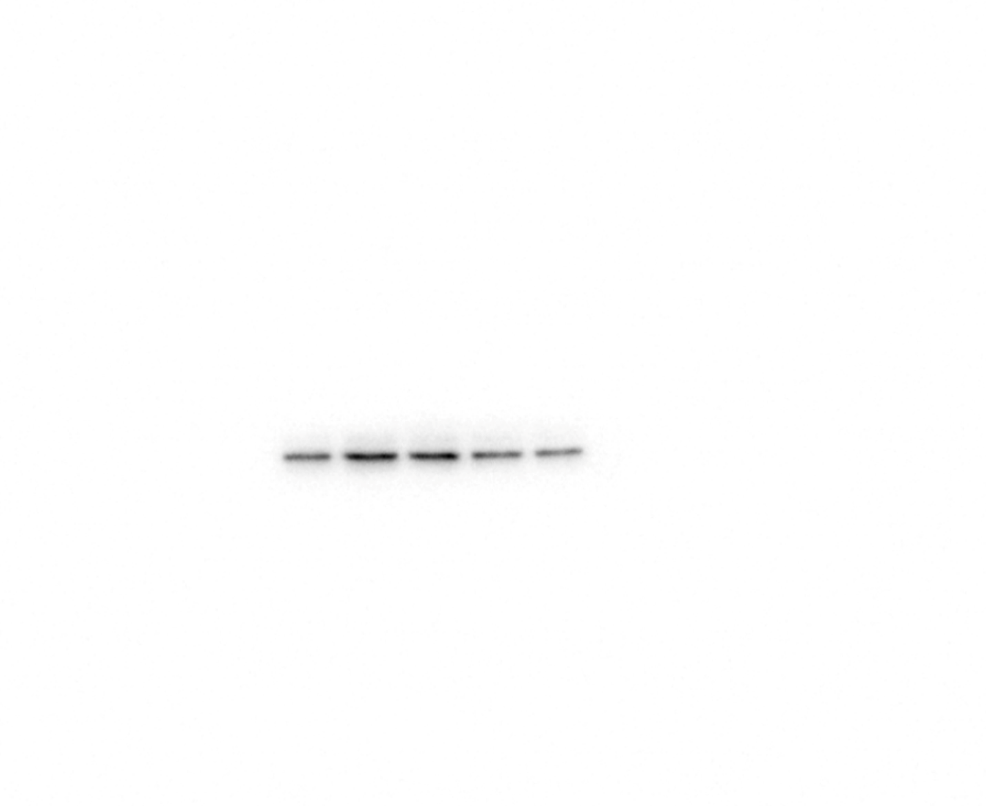


**CCI+GM 10 mg/kg**

**CCI+GM 20mg/kg**

**CCI+GM 5mg/kg**

**CCI**

**Sham**

**CD16**


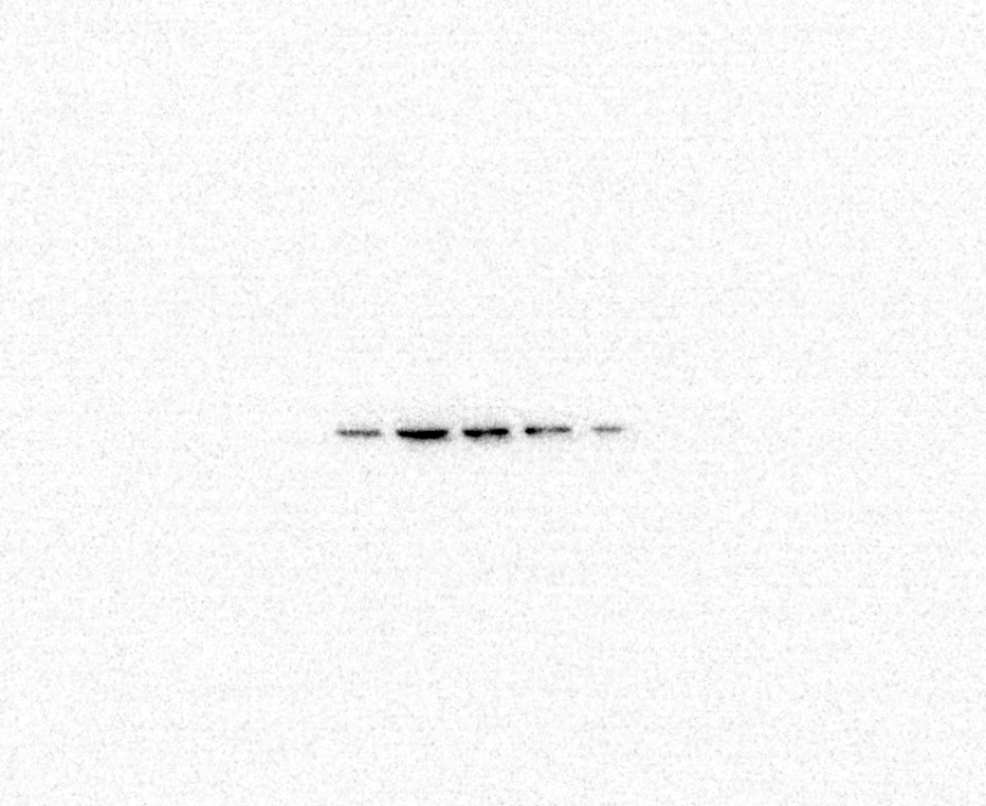


**CCI+GM 10 mg/kg**

**CCI+GM 20 mg/kg**

**CCI+GM 5mg/kg**

**CCI**

**Sham**

**CD11b**


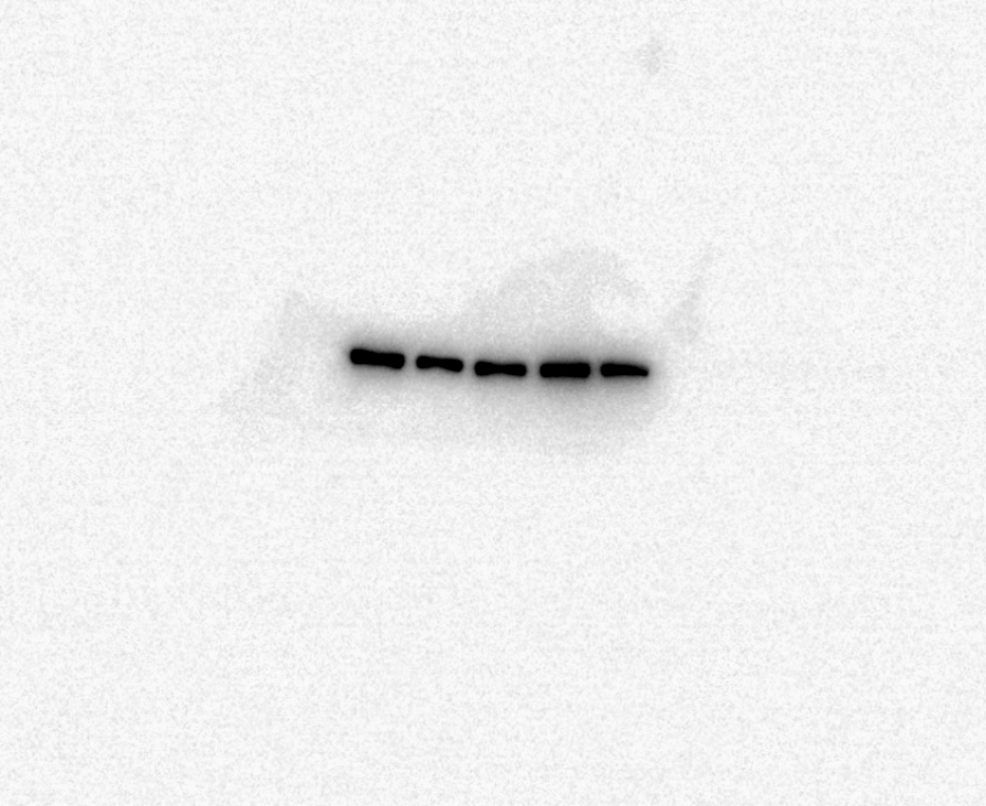


**CCI+GM 10 mg/kg**

**CCI+GM 20 mg/kg**

**CCI**

**CCI+GM 5mg/kg**

**Sham**

**β-actin**

**Figure 6**


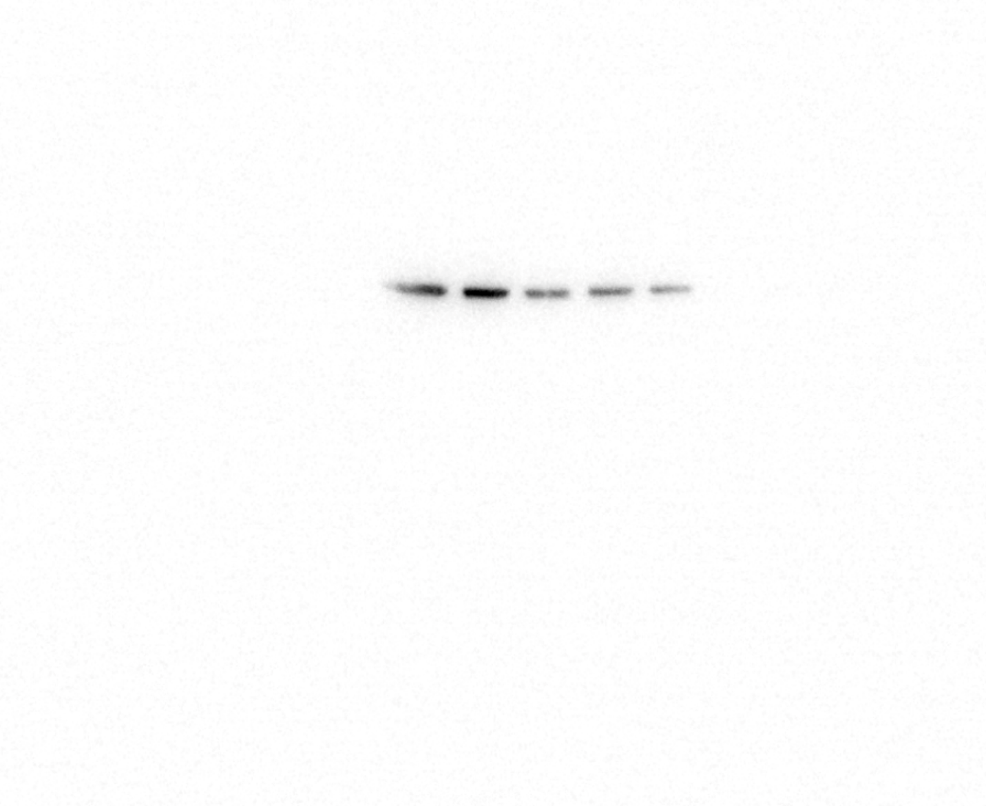

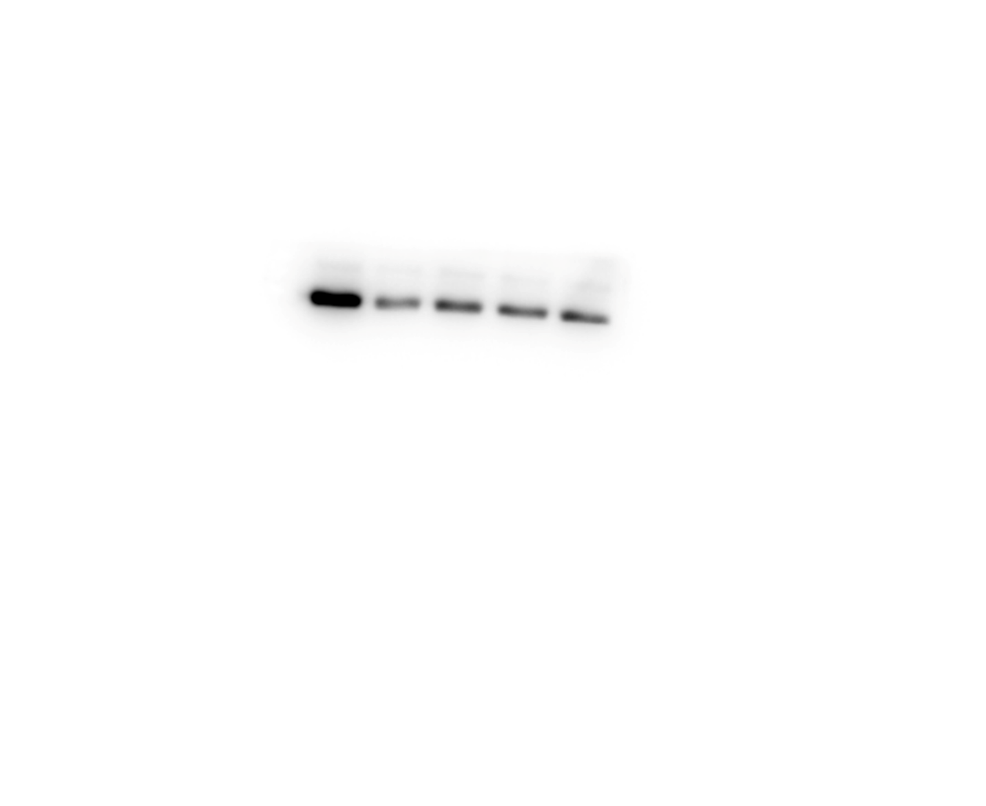

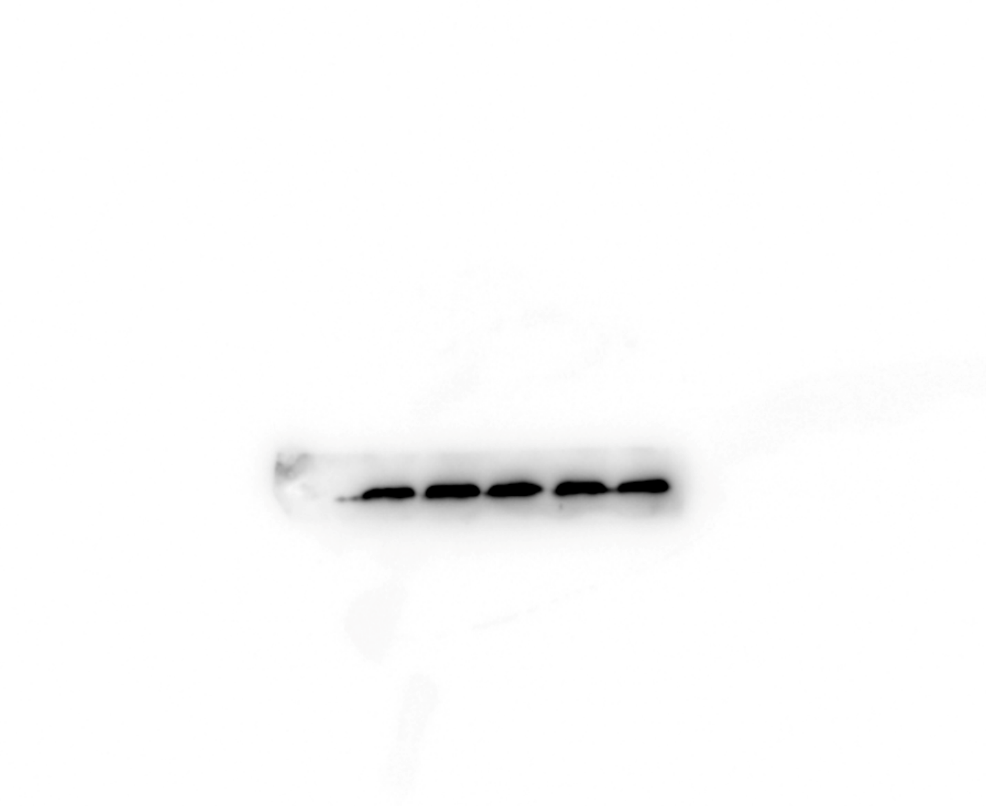


**CCI+GM 10 mg/kg**

**CCI+GM 20 mg/kg**

**CCI+GM 5mg/kg**

**CCI**

**Sham**

**p-p65**

**CCI**

**CCI+GM 20 mg/kg**

**CCI+GM 10 mg/kg**

**Sham**

**CCI+GM 5mg/kg**

**Nrf2**

**CCI+GM 20 mg/kg**

**CCI+GM 10 mg/kg**

**CCI+GM 5mg/kg**

**CCI**

**Sham**

**β-actin**

**Supplementary materials Original image of Western blot experiment in this study.**
